# Long-term exposure to a high-fat diet results in the development of glucose intolerance and insulin resistance in interleukin-1 receptor I-deficient mice

**DOI:** 10.1152/ajpendo.00297.2013

**Published:** 2013-08-06

**Authors:** Fiona C. McGillicuddy, Clare M. Reynolds, Orla Finucane, Eilish Coleman, Karen A. Harford, Christine Grant, Domenico Sergi, Lynda M. Williams, Kingston H. G. Mills, Helen M. Roche

**Affiliations:** ^1^Nutrigenomics Research Group, UCD Conway Institute, University College Dublin, Belfield, Dublin 4, Ireland;; ^2^Immune Regulation Research Group, School of Biochemistry and Immunology, Trinity College Dublin, Dublin 2, Ireland; and; ^3^Rowett Institute of Nutrition and Health, University of Aberdeen, Aberdeen, Scotland

**Keywords:** adipose inflammation, hepatic steatosis

## Abstract

Emerging evidence has demonstrated that saturated fatty acids prime pro-IL-1β production and inflammasome-mediated IL-1β activation is critical in obesity-associated insulin resistance (IR). Nonetheless, IL-1 receptor I-deficient (IL-1RI^−/−^) mice develop mature-onset obesity despite consuming a low-fat diet (LFD). With this apparent contradiction, the present study evaluated whether IL-1RI^−/−^ mice were protected against long-term (6 mo) high-fat diet (HFD)-induced IR. Male wild-type and IL-1RI^−/−^ mice were fed LFD or HFD for 3 or 6 mo, and glucose and insulin tolerance tests were performed. Adipose insulin sensitivity, cytokine profiles, and adipocyte morphology were assessed. The adipogenic potential of stromal vascular fraction was determined. Hepatic lipid accumulation and insulin sensitivity were characterized. IL-1RI^−/−^ mice developed glucose intolerance and IR after 6 mo HFD compared with 3 mo HFD, coincident with enhanced weight gain, hyperinsulinemia, and hyperleptinemia. The aggravated IR phenotype was associated with loss of adipose functionality, switch from adipocyte hyperplasia to hypertrophy and hepatosteatosis. Induction of adipogenic genes was reduced in IL-1RI^−/−^ preadipocytes after 6 mo HFD compared with 3 mo HFD. Obese LFD-IL-1RI^−/−^ mice exhibited preserved metabolic health. IL-1RI^−/−^ mice develop glucose intolerance and IR after 6 mo HFD intervention. While mature-onset obesity is evident in LFD-IL-1RI^−/−^ mice, the additional metabolic insult of HFD was required to drive adipose inflammation and systemic IR. These findings indicate an important interaction between dietary fat and IL-1, relevant to optimal metabolic health.

the spiraling increase in global rates of obesity and its associated comorbidities, including type II diabetes and nonalcoholic fatty liver disease, has provoked intense interrogation into the mechanisms by which overnutrition advances these pathological conditions. The emerging field of immunometabolism has identified a critical and intricate link between inflammation and metabolism (28). Indeed augmentation of adipose inflammation induces localized insulin resistance (IR) during obesity. This adipose IR reduces the metabolic capacity of adipose to store free fatty acid (FFA), resulting in peripheral organ lipotoxicity, culminating in systemic IR (8, 19). Obesity-associated adipose tissue inflammation in turn is likely driven by metabolic stressors, including dietary saturated fats (SFA) and endogenous ceramides (3, 11, 45). Therefore, while inflammation can directly affect metabolic processes, metabolic stressors can also activate inflammatory pathways. Localized inflammation and infiltration of proinflammatory immune cells into adipose tissue are hallmarks of obesity (15, 20, 21, 27, 31, 37, 44, 46) that contribute to IR (29, 46). Furthermore, numerous mouse strains with compromised inflammatory responses [tumor necrosis factor (TNF)^−/−^ (41), Toll-like receptor (TLR)-4^−/−^ (30, 36), and interleukin-1 receptor I (IL-1RI)^−/−^ (24)] are protected against short-term high-fat diet (HFD)-induced IR. Nevertheless, there are exceptions such as IL-6^−/−^ and IL-18^−/−^ mice that are not protected, indicating further complexities (23, 26).

The proinflammatory cytokine IL-1 has recently been recognized as a major contributor to localized adipose inflammation and IR (24, 39, 42, 45, 47). The production of bioactive IL-1β requires priming of pro-IL-1β production, usually via TLR4 activation, followed by caspase-1-mediated processing of pro- to active IL-1β (25). The Nod-like receptor family, pyrin domain-containing 3 (NLRP)-3 inflammasome complex plays an instrumental role in the activation of IL-1β during obesity (39, 42). Furthermore SFA, but not unsaturated fatty acids, can prime pro-IL-1β production, primarily via TLR4 activation, providing the substrate for the activated NLRP3 inflammasome complex (3, 32, 45). Lack of IL-1RI resulted in protection against HFD-driven IR after 3 mo feeding coupled with reduced adipose inflammation and preservation of adipose functionality (24). These findings have greatly advanced our understanding of the mechanisms by which metabolic overload with saturated fats can drive inflammation and IR and highlight a central role for IL-1 in this process.

A paradox in this field, however, is the observation that mice lacking IL-1RI develop mature-onset obesity, hyperleptinemia, and hyperinsulinemia and exhibit mild glucose intolerance when maintained on a low-fat diet (LFD) from about 4 mo of age onward (7). Most metabolic studies have been performed in young mice for a relatively short duration. The present study aimed to characterize the metabolic phenotype of long-term (6 mo) HFD exposure in IL-1RI^−/−^ mice to compare with a shorter-term (3 mo) HFD challenge or age-matched control mice maintained on LFD. Our study shows that, while protection against HFD-induced IR is evident in IL-1RI^−/−^ mice in the short term (3 mo HFD), longer-term high-fat feeding (6 mo) results in development of IR and glucose intolerance in IL-1RI^−/−^ mice with greater hepatic lipid accumulation and enhanced weight gain compared with 6 mo HFD wild-type (WT) mice. These findings demonstrate an important interaction between dietary fat and IL-1RI-mediated signals for optimal metabolic health. Complete loss of IL-1 signaling resulted in increased weight gain with age, which, when combined with a HFD metabolic insult, resulted in advanced obesity and IR.

## MATERIALS AND METHODS

### Materials

2-Deoxy-d-[1,2-^3^H(N)]glucose was purchased from Perkin-Elmer Analytical Sciences (Dublin, IRL). All other reagents unless otherwise stated were from Sigma Aldrich.

### Animals

IL-1RI^−/−^ breeding pairs, on C57BL/6 background, were purchased from Jackson Laboratories and bred in University College Dublin (UCD) for 6–10 generations under specific pathogen-free conditions. Ethical approval was obtained from the UCD Ethics Committee, and mice were maintained according to the regulations of the European Union and the Irish Department of Health. Male C57BL/6 WT and IL-1RI^−/−^ mice were fed HFD (20 kcal/100 kcal protein, 35 kcal/100 kcal carbohydrate, and 45 kcal/100 kcal fat from palm oil) or micronutrient-matched LFD (20 kcal/100 kcal protein, 70 kcal/100 kcal carbohydrate, and 10 kcal/100 kcal fat from palm oil), which were purchased from Research Diets. Mice started on diets at aged 8–10 wk for up to 6 mo (aged 8–9 mo at time of final metabolic challenge). Metabolic tests were performed after 3 mo HFD or 6 mo LFD or HFD. Before death, mice were injected with insulin (1.5 U/kg) or PBS, and after 15 min anesthetized mice were exsanguinated by cardiac puncture and killed by cervical dislocation. Adipose and liver organs were harvested.

### Intraperitoneal Glucose and Insulin Tolerance Test

Mice were fasted for 6 h and were injected intraperitoneally with 25% (wt/vol) glucose (1.5 g/kg; B. Braun Medical, Dublin, Ireland) for glucose tolerance test (GTT) or insulin (0.75 U/kg, Actrapid; Novo Nordisk) for insulin tolerance test (ITT), respectively. Glucose levels were monitored at indicated time points via tail vein blood sampling using a glucometer from Accu-Chek (Roche, Dublin, Ireland). Insulin secretory response was monitored in overnight-fasted animals, and blood samples were collected at indicated times post glucose load (1.5 g/kg ip). Insulin levels were measured by ELISA (Crystal Chem).

### Stromal Vascular Fraction Isolation and Culture

Epididymal fat pads (EAT) were minced, and adipocytes and stromal vascular fraction (SVF) cells were separated by collagenase (2 mg/ml) digestion. SVF were seeded in 12-well plates (1 × 10^6^cells/ml) in complete media (Dulbecco's modified media, 10% fetal bovine serum, and 1% penicillin/streptomycin), and after 72 h nonadherent cells were removed by adding fresh media. At *day 7*, cells were stimulated ± differentiation media (complete media supplemented with 0.5 mM 3-isobutyl-1-methylxanthine, 1 μM dexamethasone, and 1 μg/ml insulin). After 24 h cells were lysed in Tri-Reagent, and RNA was harvested.

### Flow Cytometry

SVF were filtered, blocked in PBS + 2% bovine serum albumin (BSA), and stained with the following fluorescently labeled antibodies: F4/80-FITC, CD11B-AF647/PE, and CD11C-RPE (AbD Serotec, Kidlington, UK). Unstained, single stains and fluorescence minus one were used for setting compensations and gates. Flow cytometry was performed on a Dako CyAn ADP platform (Beckman-Coulter) and analyzed using Summit v4.3 software. Cells triple positive (F4/80^+^/CD11B^+^/CD11C^+^) were classified as M1 macrophages.

### Ex Vivo Adipose Tissue Culture

Adipose explants (50 mg/ml) were cultured in complete media for 24 h. Media was harvested, and IL-6 secretion was analyzed by ELISA (Quantikine kits; R&D Systems).

### Insulin-Stimulated Glucose Uptake in Adipose Explants

Adipose explants (50 mg) were placed in PBS + 0.2% BSA before stimulation ± insulin (100 nM) for 15min. [^3^H]glucose (0.1 mM 2-deoxyglucose + 0.5 μCi/ml [^3^H]deoxyglucose) was added for 45 min. Tissue was washed, lysed in RIPA buffer, and homogenized using a tissue lyser (Qiagen, West Sussex, UK). Glucose uptake was measured by liquid scintillation counting. Fold increase in glucose uptake over basal was calculated for each individual mouse.

### General Laboratory Methods

#### Plasma analysis.

Plasma insulin, alanine aminotransferase (ALT) (Biovision), and IL-1β, IL-6, TNF-α, leptin, and adiponectin levels (Quantikine kits; R&D Systems Europe) were measured by ELISA. Plasma and liver triacylglycerol (TAG) and nonesterified fatty acid (NEFA) (Wako Chemicals) levels were measured enzymatically.

#### Real-time PCR analysis.

RNA was extracted from EAT and liver using TRI-Reagent (50 mg tissue/ml) and stored at −80°C. Single-stranded cDNA was prepared using the High-Capacity cDNA Archive Kit (Applied Biosystems, Warrington, UK). Labeled primers and probes and TaqMan Universal Mastermix were obtained from Applied Biosystems. mRNA expression was quantified by real-time PCR on an ABI 7700 Sequence Detection System (Perkin-Elmer Applied Biosystems). To control for between-sample variability, mRNA levels were normalized to 18S or GAPDH for each sample, and fold change was calculated using the relative quantification 2-(ΔΔC_t_) method as described (24).

#### Immunoblot analysis.

Protein was isolated from tissue using RIPA buffer (50 mg/ml) containing complete protease inhibitors (Roche). Protein concentration was quantified by Bradford assay (Bio-Rad Laboratories). Lysate (10 μg) was reduced, separated by SDS-PAGE, transferred to nitrocellulose membranes, blocked, and incubated overnight (at 4°C) in primary antibody. Blots were probed with antibodies to phosphorylated Akt or β-actin (Cell Signaling Technology), p21 (BD Bioscience, Oxford, UK), and CD36 (Novus Biologicals, Cambridge, UK). Blots were washed, incubated in secondary antibody, and visualized using Pierce ECL Western Blotting Substrate (Thermo Fisher Scientific).

#### Hematoxylin and eosin staining.

Adipose and liver tissue samples were fixed in formalin and paraffin embedded. Sections were prepared (5 μM) using a Leica EG1150H Machine. Hematoxylin and eosin staining was conducted using Leica Autostainer XL and Leica CV5030. Sections were mounted using DPX media (Fisher Scientific) and analyzed using a Nikon 80i transmission light microscope.

#### Citrate synthase assay.

Liver protein lysates were prepared in cell lysis buffer, and protein content was determined using a Bradford assay. Protein (10 μg) was added to a citrate synthase assay reaction, and activity was monitored every 10 s over 1.5 min. The delta absorbance was calculated, and activity in micromoles per milliliter per minute was calculated as per the manufacturer's guidelines.

### Statistical Analysis

**Fig. 1.**
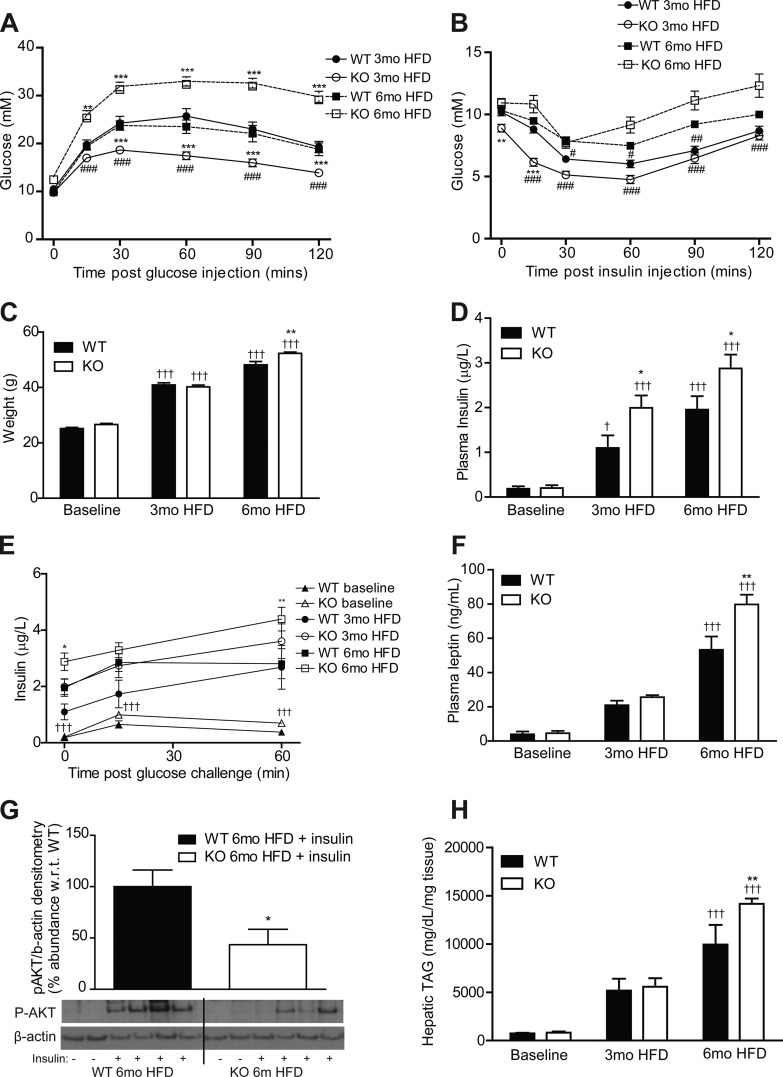
Lack of IL-1 receptor I (IL-1RI) results in development of glucose intolerance and insulin resistance after 6 mo high-fat diet (HFD). Male wild-type (WT) and IL-1 receptor I-deficient (IL-1RI^−/−^) mice (8–10 wk old) were placed on HFD for 3 or 6 mo before evaluation of glucose homeostasis after a 6-h fast by glucose tolerance test (GTT, 1.5 g/kg glucose ip, *A*) (*n* = 16–18 mice) and insulin tolerance test (ITT, 0.75 U/kg insulin ip, *B*) (*n* = 16–21). KO, knockout. *C*: weight of mice over time on HFD (*n* = 9–34). *D* and *E*: plasma levels of insulin were monitored after overnight fast (*n* = 8–20, *D*), and insulin secretion (*E*) in response to a glucose load (1.5 g/kg glucose ip) was evaluated (*n* = 6–10). *F*: plasma leptin was measured after a 6-h fast (*n* = 8–20). *G*: mice were injected ip ± insulin (1.5 U/kg) and were killed after 15 min. Insulin-induced phosphorylation of AKT in liver protein samples was analyzed by immunoblotting, and densitometry was calculated using β-actin loading control (*n* = 7). Representative blot presented. *H*: liver triacylglyceride (TAG) levels were quantified enzymatically (*n* = 8). Statistics: *P < 0.05, **P < 0.01, and ***P < 0.001, WT vs. knockout (KO); #*P* < 0.05, ##*P* < 0.01, and ###*P* < 0.001, 3 vs. 6 mo HFD; +*P* < 0.05 and +++*P* < 0.001, baseline vs. HFD.

**Fig. 2.**
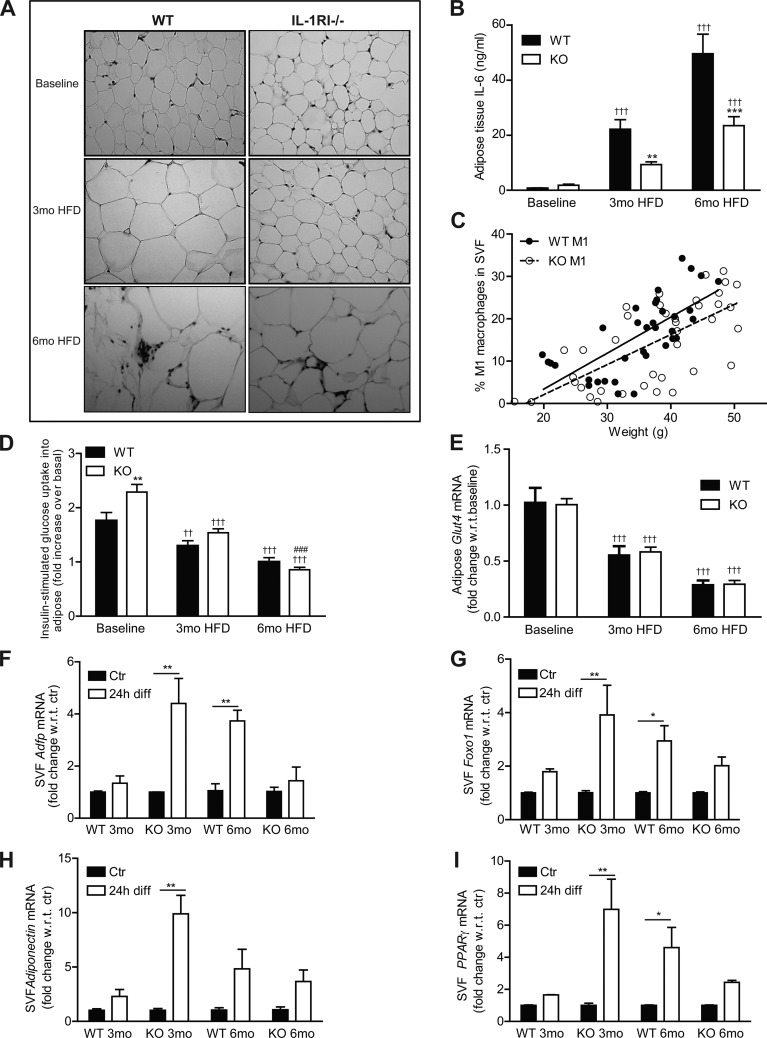
Adipose morphology of IL-1RI^−/−^ mice switched from hyperplasia after 3 mo HFD to hypertrophy after 6 mo HFD. *A*: adipocyte morphology was monitored in paraffin-embedded adipose tissue samples by hematoxylin and eosin (H&E) staining. *B*: adipose explants were cultured for 24 h in serum-containing media (50 mg/ml), and levels of IL-6 secreted in media were measured by ELISA (*n* = 8). *C*: percentage of M1 macrophages (F480^+^/CD11B^+^/CD11C^+^) in the stromal vascular fraction (SVF) of adipose tissue was monitored by flow cytometry and correlated to the weight of individual mice (*P* < 0.001, WT *r*^2^ = 0.49; IL-1RI^−/−^ *r*^2^ = 0.46). *D*: insulin (100 nM)-stimulated [^3^H]glucose uptake in adipose explants was monitored immediately after death. Fold increase in [^3^H]glucose transport in response to insulin over basal (noninsulin stimulated) for each individual mouse was calculated and is presented (*n* = 8). *E*: adipose tissue RNA was harvested, and mRNA levels of GLUT4 were measured by real-time PCR analysis (*n* = 4–6). Statistics: **P < 0.01 and ***P < 0.001, WT vs. KO; ###*P* < 0.001, 3 vs. 6 mo HFD; ++*P* < 0.01 and +++*P* < 0.001, baseline vs. HFD. *F*–*I*: adipose-derived SVF cells were isolated from mice after 3 or 6 mo HFD and were cultured for 7 days to enrich for preadipocytes. Cells were then incubated ± differentiation media for 24 h, and mRNA expression of adipose differentiation-related protein (Adfp, *F*), forkhead box protein O1 (FOXO1, *G*), adiponectin (*H*), and peroxisome proliferator-activated receptor (PPAR)-γ (*I*) was assessed by real-time PCR. Expression was normalized for GAPDH, and fold change in expression relative to individual nondifferentiated control is presented [*P < 0.05 and **P < 0.01, control (Ctr) vs. differentiated (diff 24 h), *n* = 4].

**Fig. 3.**
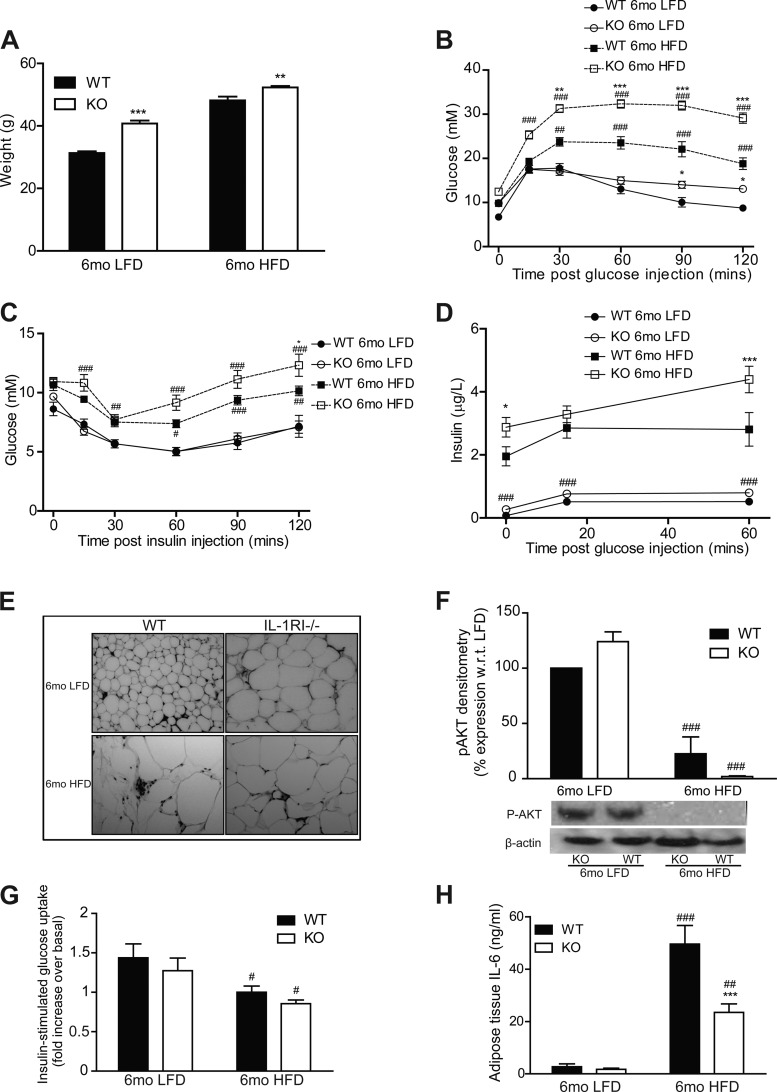
IL-1RI^−/−^ mice develop mature-onset obesity on a low-fat diet (LFD) but have preserved glucose homeostasis and negligible adipose tissue inflammation. *A*: weight of male WT and IL-1RI^−/−^ mice (8–10 wk old) after 6 mo on HFD or LFD (*n* = 9–34). Glucose homeostasis was monitored by GTT (*n* = 13–31, *B*) and ITT (*n* = 9–23, *C*). *D*: insulin secretion in response to a glucose load (1.5 g/kg glucose ip) was evaluated after 6 mo HFD or 6 mo LFD (*n* = 6–10). *E*: adipocyte morphology was monitored in paraffin-embedded adipose tissue samples by H&E staining. *F*: adipose explants were stimulated ± insulin (100 nM) for 60 min ex vivo, and levels of phosphorylated AKT and β-actin were measured by immunoblot analysis (*n* = 3). *G*: insulin (100 nM)-stimulated [^3^H]glucose uptake in adipose explants was monitored immediately after death. Fold increase in [^3^H]glucose transport in response to insulin over basal (noninsulin stimulated) for each individual mouse was calculated and is presented (*n* = 6–11). *H*: IL-6 secretion levels from adipose explants was measured after 24 h incubation in complete media (*n* = 8). Statistics: #*P* < 0.05, ##*P* < 0.01, and ###*P* < 0.001, HFD vs. LFD groups; *P < 0.5, **P < 0.01, and ***P < 0.001, WT vs. KO.

**Fig. 4.**
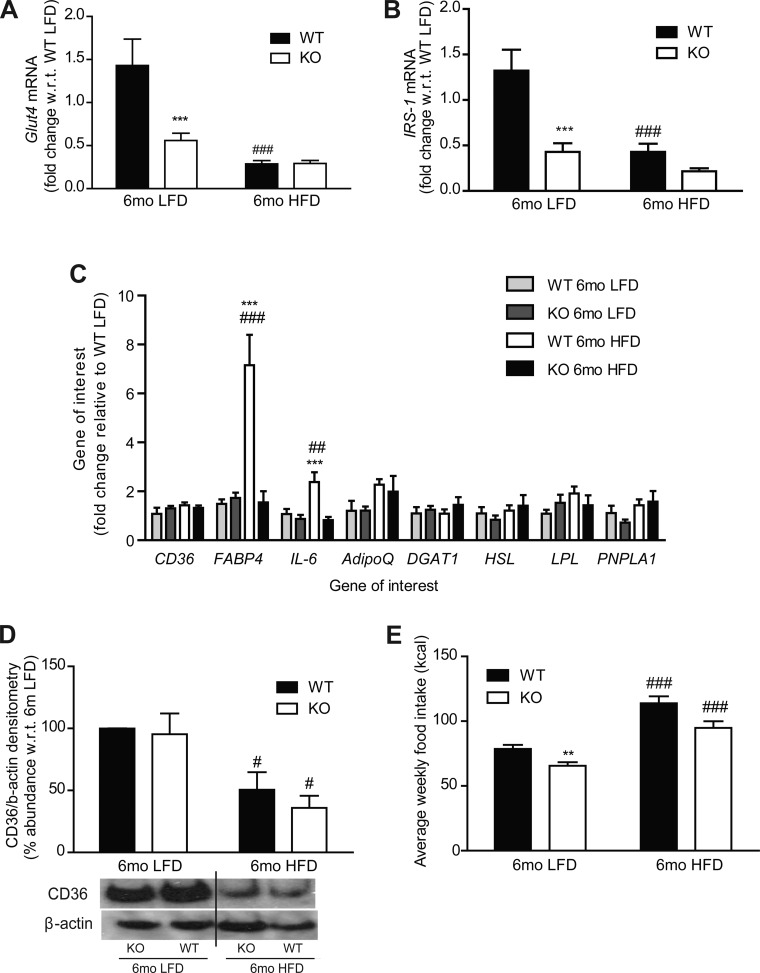
Adipose gene and protein expression analysis after 6 mo LFD and HFD in WT and IL-1RI^−/−^ mice. *A*–*C*: adipose tissue RNA was harvested, and mRNA levels of Glut4 (*A*), insulin receptor substrate (IRS)-1 (*B*), CD36 (*C*), fatty acid-binding protein (FABP)-4, IL-6, diglyceride acyltransferase (DGAT), adiponectin (adipoQ), lipoprotein lipase (LPL), hormone sensitive lipase (HSL), and patatin-like phospholipase domain containing 1 (PNPLA1) were measured by real-time PCR (***P < 0.001, WT vs. KO; ##*P* < 0.01 and ###*P* < 0.001, LFD vs. HFD, *n* = 4–8). *D*: protein levels of CD36 and β-actin loading control were measured in adipose by immunoblot analysis; representative blot and densitometry analysis are shown (#*P* < 0.05, LFD vs. HFD, *n* = 3). *E*: average weekly caloric intake per mouse on LFD and HFD (**P < 0.01, WT vs. KO; ###*P* < 0.001, LFD vs. HFD). Intake measured weekly and averaged over 6 wk (HFD) or 25 wk (LFD), *n* = 3 cages/group.

**Fig. 5.**
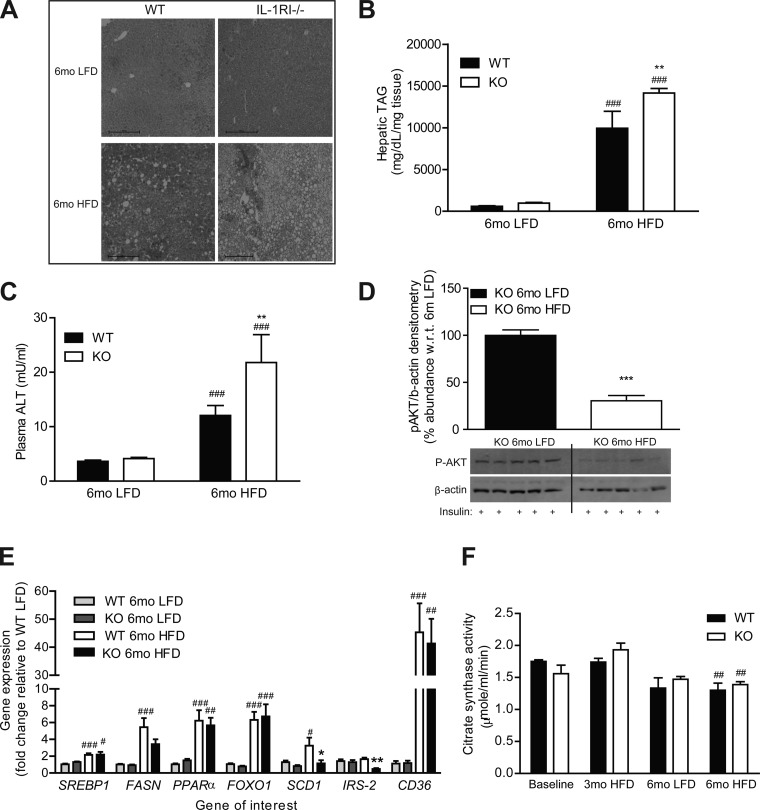
Characterization of hepatic lipotoxicity and insulin sensitivity after 6 mo intervention with either LFD or HFD. *A*: liver morphology of WT and IL-1RI^−/−^ mice was monitored after 6 mo LFD and HFD by H&E staining. *B*: liver triacylglyceride (TAG) levels were quantified enzymatically (*n* = 8). *C*: plasma alanine aminotransferase (ALT) levels were monitored by ELISA (*n* = 5–14). *D*: mice were injected ip ± insulin (1.5 U/kg) and were killed after 15 min. Insulin-induced phosphorylation of AKT in liver protein samples was analyzed by immunoblotting, and densitometry was calculated relative to β-actin loading control (*n* = 10–13). Representative blot presented. *E*: liver mRNA was isolated and reverse transcribed before real-time PCR analysis of lipogenic genes (*n* = 12–14). 18S was used as an internal control, and genes are expressed relative to 6 mo WT LFD group. *F*: citrate synthase activity was monitored in WT and IL-1RI^−/−^ liver lysates after LFD or HFD and compared with baseline levels as a biomarker of mitochondrial function (*n* = 6). Statistics: #*P* < 0.05, ##*P* < 0.01, and ###*P* < 0.001, LFD vs. HFD; *P < 0.05, **P < 0.01, and ***P < 0.001, WT vs. KO.

Data are reported as means ± SE. For GTT/ITT studies with multiple time points, we performed two-way repeated-measures ANOVA to test for differences in means between WT and IL-1RI^−/−^ groups. When ANOVA was significant, post hoc Bonferroni corrected *t*-tests were applied. For comparison of data between two groups at a single time point, unpaired *t*-tests were performed. GraphPad Prism 5 (GraphPad Software, San Diego, CA) was used for statistical analyses. Statistical significance is presented Figs. 1–5.

## RESULTS

### Loss of Protection Against HFD-Induced IR in IL-1RI^−/−^ after 6 mo Dietary Intervention

IL-1RI^−/−^ mice became glucose intolerant and exhibited enhanced weight gain compared with WT after 6 mo HFD despite improved glucose homeostasis after 3 mo HFD, as previously described (24) (Fig. 1, *A*–*C*). IL-1RI^−/−^ mice became progressively more insulin resistant between 3 and 6 mo HFD by ITT with a comparable glucose-lowering response as WT after 6 mo HFD (Fig. 1*B*). The progressive development of IR was also evident in weight-matched mice; IL-1RI^−/−^ mice weighing 35–45 g exhibited enhanced glucose clearance during GTT, whereas 45–55 g IL-1RI^−/−^ mice exhibited increased hyperglycemia during GTT compared with weight-matched WT (data not shown). Fasting IL-1RI^−/−^ mice were hyperinsulinemic compared with WT after 3 and 6 mo HFD (Fig. 1*D*). Furthermore, after a glucose challenge, HFD-fed IL-1RI^−/−^ mice exhibited an increased insulin secretion response compared with WT (Fig. 1*E*). Plasma leptin was also elevated in IL-1RI^−/−^ mice after 6 mo HFD (Fig. 1*F*) while plasma TAG and NEFA increased equivalently in both strains (Table 1). Feeding HFD also increased plasma TNF-α, IL-6, and adiponectin levels in both strains, albeit not significantly (Table 1). Hepatic insulin sensitivity was evaluated after intraperitoneal injection of insulin before death. Phosphorylated (p)-AKT was reduced in 6 mo HFD IL-1RI^−/−^ livers compared with WT (Fig. 1*G*). Hepatic TAG accumulation increased progressively on HFD (Fig. 1*H*) with significantly elevated levels in IL-1RI^−/−^ mice compared with WT after 6 mo HFD.

**Table 1.** Plasma metabolic profile

	WT 6 mo LFD	KO 6 mo LFD	WT 6 mo HFD	KO 6 mo HFD
TAG, mmol/l	0.62 ± 0.06	0.65 ± 0.04	1.44 ± 0.14^###^	1.25 ± 0.09^###^
NEFA, mmol/l	0.53 ± 0.03	0.74 ± 0.09	1.85 ± 0.14^###^	1.58 ± 0.17^###^
TNF-α, pg/ml	13.5 ± 4.6	5.9 ± 1.3	22.5 ± 7.1	15.5 ± 6.2
IL-6, pg/ml	17.6 ± 5.4	11.7 ± 4.5	34.3 ± 8.5	31.9 ± 9.1
Insulin, μg/l	0.07 ± 0.02	0.27 ± 0.09	1.95 ± 0.3^###^	2.87 ± 0.3*^###^
Leptin, ng/ml	13.34 ± 3.36	40.91 ± 13.36*	53.23 ± 7.75^###^	79.72 ± 5.72*^##^
Adiponectin, ng/ml	5,488 ± 678	4,902 ± 503	6,387 ± 503	5,296 ± 369

Values are means ± SE; *n* = 8 mice in each group. WT, wild type; LFD, low-fat diet; KO, knockout; TAG, triacylglycerol; NEFA, nonesterified fatty acid; TNF-α, tumor necrosis factor-α. Plasma was isolated from WT and IL-1 receptor I-deficient mice by cardiac puncture, and levels of metabolic markers were analyzed enzymatically (###*P* < 0.001, LFD vs. HFD; *P < 0.05, WT vs. KO).

### Adipose Morphology Switches from Hyperplasia after 3 mo HFD to Hypertrophy after 6 mo HFD Coincident with Reduced Adipogenic Potential of Stromal Vascular Cells

To further understand the mechanisms responsible for the delayed development of insulin resistance in IL-1RI^−/−^ mice on HFD, we assessed adipose morphology and functionality. Adipocyte morphology revealed a switch in IL-1RI^−/−^ mice from a hyperplastic phenotype after 3 mo HFD to a hypertrophic phenotype after 6 mo HFD (Fig. 2*A*) while adipocyte hypertrophy was already evident after 3 mo HFD in WT mice. Adipose inflammation was assessed by monitoring cytokine secretion and M1 macrophage infiltration into adipose tissue. Adipose explant IL-6 secretion increased progressively with HFD in both strains, but levels remained significantly lower in IL-1RI^−/−^ explants (Fig. 2*B*). Recruitment of M1 macrophages (F4/80^+^/CD11b^+^/CD11c^+^) did not differ between genotypes and significantly correlated to weight irrespective of genotype (Fig. 2*C*). Insulin-stimulated [^3^H]glucose uptake into adipose explants was more efficient in IL-1RI^−/−^ mice at baseline, but negligible uptake was observed after 6 mo HFD in both strains (Fig. 2*D*). GLUT4 (Fig. 2*E*) and insulin receptor substrate (IRS)-1 (data not shown) mRNA levels also progressively decreased on HFD in both strains.

To understand if altered adipose morphology reflected different adipogenic potential of preadipocytes, we characterized the induction of adipogenic genes in SVF cells after incubation in differentiation media for 24 h. SVF were grown in culture for 7 days before incubation in differentiation media to remove any nonadherent cells, allowing for “preadipocyte” enrichment. Interestingly, after 3 mo, HFD IL-1RI^−/−^ SVF had the greatest response to adipogenic media with increased expression of adipogenic-related genes, including adipose differentiation-related protein, forkhead box protein O1 (FOXO1), adiponectin, and peroxisome proliferator-activated receptor (PPAR)-γ (Fig. 2, *F*–*I*). However, this response was blunted in IL-1RI^−/−^ SVF after 6 mo HFD.

### IL-1RI^−/−^ Mice Develop Spontaneous Obesity on a LFD but Exhibit Preservation of Adipose Functionality and Metabolic Health

Consistent with the data reported by Garcia et al. (7), IL-1RI^−/−^ mice developed mature-onset obesity after 6 mo LFD (Fig. 3*A*). Despite this weight gain, glucose homeostasis was only moderately impaired (Fig. 3, *B* and *C*). Furthermore insulin secretion was significantly lower in obese LFD-IL-1RI^−/−^ mice compared with HFD-IL-1RI^−/−^ mice (Fig. 3*D*). The additional metabolic insult of HFD in mature IL-1RI^−/−^ animals was therefore required to drive IR despite the obese phenotype of LFD mice. Fasting plasma insulin levels were marginally increased in 6 mo LFD-IL-1RI^−/−^ mice compared with 6 mo LFD WT (*P* = 0.07), and enhanced insulin secretion in response to a glucose load was evident (area under the curve WT vs. knockout LFD, *P* = 0.0528) (Fig. 3*D*). The obese phenotype in IL-1RI^−/−^ mice on LFD was also associated with the development of adipocyte hypertrophy (Fig. 3*E*). Nonetheless, insulin-stimulated induction of p-AKT (Fig. 3*F*) and [^3^H]glucose uptake into explants (Fig. 3*G*) were significantly higher in 6 mo LFD adipose compared with 6 mo HFD, indicative of preserved functionality. Adipose mRNA expression of GLUT4 and IRS-1 (Fig. 4, *A* and *B*) was attenuated in IL-1RI^−/−^ adipose after both LFD and HFD, which may reflect the hypertrophic state of the adipocytes. Adipose IL-6 secretion (both strains) (Fig. 3*H*) and IL-6 mRNA (WT only) (Fig. 4*C*) were significantly higher in HFD adipose compared with LFD, indicative that a HFD is required to stimulate adipose inflammation.

Little change in genes involved in adipose lipolysis, fatty acid metabolism, and fatty acid transport (CD36, diglyceride acyltransferase, adiponectin, lipoprotein lipase, hormone-sensitive lipase, patatin-like phospholipase domain containing 1) was observed (Fig. 4*C*). However, fatty acid-binding protein 4 (FABP4) mRNA expression was significantly higher in WT adipose tissue after 6 mo HFD compared with IL-1RI^−/−^ adipose tissue (Fig. 4*C*). Adipose expression of the fatty acid transporter CD36 was significantly reduced in both strains following 6 mo HFD but not in LFD groups, despite negligible change at the mRNA level (Fig. 4*D*). Despite increased weight gain in IL-1RI^−/−^ mice after both 6 mo LFD and HFD, caloric intake was slightly lower in IL-1RI^−/−^ mice compared with WT (Fig. 4*E*).

### Enhanced Hepatic Lipotoxicity and Reduced Insulin Sensitivity after 6 mo HFD, but not LFD, in IL-1RI^−/−^ Mice

Increased hepatic accumulation of TAG and plasma ALT levels was observed after 6 mo HFD in IL-1RI^−/−^ mice compared with WT (Fig. 5, *A*–*C*). IL-1RI^−/−^ mice fed a LFD for 6 mo exhibited normal liver morphology, hepatic TAG concentrations, and plasma ALT levels despite their obese phenotype (Fig. 5, *A*–*C*). In terms of hepatic insulin sensitivity, p-AKT levels were preserved in 6 mo LFD IL-1RI^−/−^ livers compared with HFD, indicative of preserved peripheral insulin sensitivity (Fig. 5*D*). Hepatic fatty acid synthase (FASN) and stearoyl-CoA-desaturase (SCD)-1 mRNA expression increased significantly after 6 mo HFD in WT but not in IL-1RI^−/−^ mice compared with 6 mo LFD WT, whereas IRS-2 expression was significantly reduced in 6 mo HFD IL-1RI^−/−^ livers compared with 6 mo HFD WT (Fig. 5*E*). Real-time PCR also revealed equivalent induction in lipogenic genes, sterol response element-binding protein 1, PPARα, and FOXO1 and in the fatty acid transporter CD36 in WT and IL-1RI^−/−^ livers after 6 mo HFD but not LFD (Fig. 5*E*). Hepatic mitochondrial activity was assessed by citrate synthase assay and revealed significant reduction in activity with age in both genotypes regardless of diet (Fig. 5*F*).

## DISCUSSION

There is increasing evidence that inflammatory responses, especially those mediated by IL-1β and the NLRP3 inflammasome, play a central role in HFD-induced IR. Our current study advances these findings into longer-term feeding studies and highlights the complexities of IL-1 signaling in metabolism. We demonstrate that IL-1RI^−/−^ mice maintained on HFD for 6 mo develop glucose intolerance and insulin resistance coupled with a switch in adipose morphology from hyperplasia after 3 mo HFD to hypertrophy after 6 mo HFD, augmentation of adipose inflammation, loss of adipose insulin sensitivity, hepatosteatosis, and hepatic IR. This study, and previous reports (2, 7), demonstrate that chow-fed IL-1RI^−/−^ mice develop mature-onset obesity, hyperleptinemia, and hyperinsulinemia. Nonetheless, obese LFD-fed IL-1RI^−/−^ mice exhibited only moderate glucose intolerance, negligible adipose inflammation, and preservation of adipose insulin sensitivity compared with age-matched HFD counterparts. The additional metabolic insult of HFD, and resulting advanced obese state, was necessary for loss of functionality of adipose tissue and development of systemic IR.

The switch in adipose tissue morphology observed in IL-1RI^−/−^ mice between 3 and 6 mo HFD may have been driven by numerous mechanisms. IL-1 is a potent inhibitor of adipogenesis (18), and induction of IL-1 in WT mice on HFD may prevent preadipocyte maturation and drive adipocyte hypertrophy, an effect that is absent in IL-1RI^−/−^ mice in early life. The capacity of preadipocytes to undergo adipogenesis, however, decreases with maturity (33, 40), coincident with reduced expression of transcription factors CEBP1α (14) and PPARγ (13) and the development of cellular senescence within the preadipocyte population (40). IL-6 is implicated in development of cellular senescence (17), and induction of IL-6 with HFD may accelerate aging in adipose tissue. In agreement with this hypothesis, we found adipose tissue expression of the senescent marker p21 increased after 6 mo HFD in both genotypes compared with LFD groups (data not shown). Furthermore, we demonstrate that induction of adipogenic genes within SVF cells is highest after 3 mo HFD in IL-1RI^−/−^ cells and is blunted after 6 mo HFD, indicating that any enhanced adipogenic potential is lost in the long term. Reduced de novo adipogenesis coupled with increased FFA storage demand due to prolonged exposure to HFD may account for the switch in adipose tissue morphology from hyperplasia to hypertrophy in IL-1RI^−/−^ mice.

Adipose tissue inflammation is a key mechanism driving localized IR, which precedes systemic IR (9, 12, 37). We recently demonstrated that reduced adipose inflammation correlated with amelioration of HFD-induced IR in IL-1RI^−/−^ mice after 3 mo HFD (24). In the current study, adipose IL-6 secretion was further increased after 6 mo HFD in both genotypes, although levels remained significantly lower from IL-1RI^−/−^ explants. IL-6 secretion after 6 mo HFD in IL-1RI^−/−^ mice had reached levels equivalent to WT following 3 mo HFD. This level may represent a critical threshold that was sufficient to drive adipocyte IR and loss of adipose functionality. Notably, obese LFD-derived IL-1RI^−/−^ adipose explants secreted negligible levels of IL-6, demonstrating the necessity of a HFD insult to drive the localized inflammation of obesity.

Hepatic lipid accumulation and plasma ALT levels were significantly higher in IL-1RI^−/−^ mice after 6 mo HFD compared with WT and may account for the reduced glucose tolerance in IL-1RI^−/−^ mice at this time point. This enhanced hepatic lipid accumulation may have been attributable to the greater weight gain in IL-1RI^−/−^ mice and loss of efficient storage capacity of the expanded adipose tissue, since key hepatic lipogenic markers, FASN and SCD, were not induced in 6 mo HFD IL-1RI^−/−^ compared with their WT counterparts. Reduced levels of insulin-stimulated p-AKT were also observed after 6 mo HFD in IL-1RI^−/−^ livers compared with both WT 6 mo HFD and knockout 6 mo LFD, indicative of reduced hepatic insulin sensitivity. Lack of enhanced adipose FABP4 and reduced adipose CD36 expression may have contributed to shuttling of fatty acids to peripheral tissues, contributing to hepatic lipid accumulation in IL-1RI^−/−^ mice. IL-6^−/−^ mice also develop hepatosteatosis, systemic IR (23), and mature-onset obesity (43). Given that IL-1 plays an important role in the regulation of IL-6 (24), it is feasible that the underlying mechanism driving these phenotypes is similar between strains. The hyperinsulinemic state in long-term HFD IL-1RI^−/−^ mice may also have stimulated greater lipid accumulation (1, 35) and enhanced lipotoxicity. Hepatic lipid accumulation and IR was not observed in livers of spontaneously obese LFD-fed IL-1RI^−/−^ mice, which have significantly lower plasma insulin levels compared with HFD-fed counterparts. Preservation of hepatic insulin sensitivity in 6 mo LFD-fed mice probably accounts for their improved metabolic profile compared with HFD-fed mice. Notably, obese 6 mo LFD-fed IL-1RI^−/−^ mice weigh significantly less than 6 mo HFD-fed IL-1RI^−/−^ mice (40 vs. 52 g). Thus differences in metabolic phenotypes may be attributable to the more advanced obese state in the HFD group.

The development of systemic IR in IL-1RI^−/−^ mice after 6 mo HFD coincides with the time point at which chow-fed counterparts develop mature-onset obesity (7). Conversely, IL-1 receptor antagonist^−/−^ (IL-1Ra^−/−^) mice exhibit reduced weight gain (22, 38). The degree to which IL-1 regulates weight, however, must be very subtle. IL-1RI^−/−^ mice are hyperleptinemic, but overt hyperphagia was not evident in our study or previously observed (7), nor is reduced food intake observed in lean IL-1Ra^−/−^ mice (38). Enhanced leptin levels in IL-1RI^−/−^ mice may merely reflect increased adipose tissue mass. Reduced locomotor activity is a feature of both IL-1RI^−/−^ (7) and IL-1β^−/−^ (16) mice. No difference in overall energy expenditure was observed in IL-1RI^−/−^ mice, although a reduction in fat-to-carbohydrate oxidation ratio was observed (7). The subtle, long-term accumulation of reduced activity and switch in energy substrate utilization may account for the gradual weight gain in IL-1RI^−/−^ mice, which eventually manifests as mature-onset obesity.

The IR phenotype of IL-1RI^−/−^ after prolonged HFD and the intrinsic capacity of IL-1 to regulate weight warrants caution with the long-term use of IL-1-neutralizing therapies for the treatment of IR. Indeed, the IL-6 receptor-inhibiting monoclonal antibody tocilizumab has resulted in increased weight gain and hyperlipidemia in humans (5), and there are analogous signaling and metabolic phenotypes associated with ablation of IL-1 and IL-6. Notably, IL-18^−/−^ (26) and signal transducer and activator of transcription (STAT) 3^−/−^ (6) mice also develop spontaneous obesity, and impaired STAT3 phosphorylation was proposed to underlie this phenotype in IL-18^−/−^ mice. Recent findings by Henao-Mejia et al. have demonstrated that inflammasome deficiency (NLRP6^−/−^, ASC^−/−^, and IL-18^−/−^) resulted in significant changes in the gut microbiome, resulting in exaggerated hepatosteatosis and inflammation when maintained on a methionine-choline-deficient diet (MCDD), a phenotype that was transferrable to WT mice upon cohousing (10). Furthermore, ASC^−/−^ mice gained more weight and steatosis on HFD, an effect that was also transferrable to cohoused WT mice. IL-1RI^−/−^ mice were not prone to MCDD-induced hepatic injury (10), but the long-term effects of HFD were not examined. It is plausible that changes in the gut microbiome of IL-1RI^−/−^ mice may be contributing to exaggerated weight gain, and future studies are warranted.

The NLRP3 inflammasome complex is responsible for processing of both pro-IL-1 and pro-IL-18; nonetheless, NLRP3^−/−^ mice are not prone to the development of spontaneous obesity (42), indicative that the NLRP3-inflammasome complex may not be responsible for processing of central pro-IL-1β or pro-IL-18 that likely maintains weight homeostasis. As NLRP3 specifically recognizes host-derived danger-associated molecular patterns, including metabolic derivatives/stressors (4, 34), specific targeting of NLRP3 may represent the most viable therapeutic option to block IL-1 processing locally within obese adipose tissue while simultaneously preserving the beneficial effects of central IL-1 on weight homeostasis.

This study has highlighted the importance of maintaining physiological levels of IL-1 signaling to sustain healthy metabolism. Excessive induction of IL-1 during high-fat feeding drives adipose tissue inflammation and IR while complete abrogation of IL-1 results in excessive weight gain with maturity that, when combined with a high-fat insult, results in advanced obesity and systemic IR. This study has a number of potential limitations, including the use of only male mice, use of nonsibling controls, and use of whole body knockout mice. It is difficult to decipher the underlying mechanisms that contribute to the development of IR in whole body IL-1RI^−/−^ mice; whether lack of central, hepatic, or hematopoietic IL-1 is the main perpetrator remains to be addressed. This study has, however, indicated the importance of the gene-nutrient environment; in the absence of a high-fat insult, IL-1RI^−/−^ mice, who are genetically prone to obesity, exhibit only mild glucose intolerance and negligible adipose inflammation. The additional metabolic trigger of HFD is too great an insult in these mice, and advanced obesity, glucose intolerance, adipose tissue inflammation, and IR ensue.

## GRANTS

This work was supported by Science Foundation Ireland PI Programme (06/IM.1/B105 and 11/PI/1119) (H. M. Roche). F. McGillicuddy is jointly funded by Science Foundation Ireland, the Health Research Board, and the Wellcome Trust (097311/Z/11/Z) under the SFI-HRB-Wellcome Trust Biomedical Research Partnership. L. Williams is supported by the Scotish government's rural and environment science and analytical services division.

## DISCLOSURES

Dr. McGillicuddy and Professor Roche are the guarantors of this work, had full access to all the data, and take full responsibility for the integrity of data and the accuracy of data analysis. The authors have no conflict of interest in relation to this manuscript.

## AUTHOR CONTRIBUTIONS

Author contributions: F.C.M., K.H.M., and H.M.R. conception and design of research; F.C.M., C.M.R., O.F., E.C., K.A.H., C.G., D.S., and L.M.W. performed experiments; F.C.M. analyzed data; F.C.M. and H.M.R. interpreted results of experiments; F.C.M. prepared figures; F.C.M. drafted manuscript; F.C.M., L.M.W., K.H.M., and H.M.R. edited and revised manuscript; F.C.M., L.M.W., and H.M.R. approved final version of manuscript.
